# MULTIMORBIDITY RESEARCH: CHALLENGES IN RECRUITMENT AND RETENTION OF OLDER ADULTS

**DOI:** 10.1093/geroni/igae098.3018

**Published:** 2024-12-31

**Authors:** Binu Koirala, Chitchanok Benjasirisan, Arum Lim, Jordan Tebay, Cheryl Dennison Himmelfarb, Patricia Davidson

**Affiliations:** Johns Hopkins University, Baltimore, Maryland, United States; Johns Hopkins University, Baltimore, Maryland, United States; Johns Hopkins University, Baltimore, Maryland, United States; Johns Hopkins University, Baltimore, Maryland, United States; Johns Hopkins University, Baltimore, Maryland, United States; University of Wollongong, Wollongong, New South Wales, Australia

## Abstract

With an aging population, medical advancement, and longer life expectancies, the prevalence of multimorbidity is increasing. This demands high-quality health research to tailor and develop effective multimorbidity prevention and management programs. Recruitment and retention are always a concern in research, and it could be more challenging and time-consuming for older adults. This session aims to identify and discuss challenges for the recruitment and retention of older adults with multimorbidity in health research from the learnings of our projects. We conducted four studies among older adults living with multimorbidity: two experience-based co-design studies to understand disease trajectory and symptom burden (n=9 patients; n=2 family caregivers; n=11 healthcare providers) and gender differences on care preferences and outcome (n=24 patients; n=7 family caregivers;15 healthcare providers); a quasi-experimental study implementing a care-coordination and symptom management (COORDINATE) program (n=25); and a cohort study in gender differences in health outcomes (n=353). Recruiting and retaining older adults in research could be affected by multi-level factors including social and structural determinants of health. These challenges can be discussed under 1) individual factors (participants’ biological, physical, psychological, social status, work commitments, financial strain/availability of research instruments such as phones and capacity to use digital devices and electronic portals), research-related factors (availability of the research team, effective communication techniques, and survey design) and system-level factors (limited universal definition of multimorbidity, health and technology access). Our study demonstrated the feasibility of research among older adults living with multimorbidity, yet challenges exist in recruitment and retention and require significant resources and planning.

